# Persuasive Systems Design Trends in Coronary Heart Disease Management: Scoping Review of Randomized Controlled Trials

**DOI:** 10.2196/49515

**Published:** 2024-06-19

**Authors:** Eunice Eno Yaa Frimponmaa Agyei, Akon Ekpezu, Harri Oinas-Kukkonen

**Affiliations:** 1 Oulu Advanced Research on Service and Information Systems Faculty of Information Technology and Electrical Engineering University of Oulu Oulu Finland

**Keywords:** coronary heart disease, persuasive systems design, behavior change, randomized controlled trial, RCT, controlled trials, heart, CHD, cardiovascular

## Abstract

**Background:**

Behavior change support systems (BCSSs) have the potential to help people maintain healthy lifestyles and aid in the self-management of coronary heart disease (CHD). The Persuasive Systems Design (PSD) model is a framework for designing and evaluating systems designed to support lifestyle modifications and health behavior change using information and communication technology. However, evidence for the underlying design principles behind BCSSs for CHD has not been extensively reported in the literature.

**Objective:**

This scoping review aims to identify existing health BCSSs for CHD, report the characteristics of these systems, and describe the persuasion context and persuasive design principles of these systems based on the PSD framework.

**Methods:**

Using the PRISMA-ScR (Preferred Reporting Items for Systematic Reviews and Meta-Analyses Extension for Scoping Reviews) guidelines, 3 digital databases (Scopus, Web of Science, and MEDLINE) were searched between 2010 to 2022. The major inclusion criteria for studies were in accordance with the PICO (Population, Intervention, Comparison, and Outcome) approach.

**Results:**

Searches conducted in the databases identified 1195 papers, among which 30 were identified as eligible for the review. The most interesting characteristics of the BCSSs were the predominant use of primary task support principles, followed by dialogue support and credibility support and the sparing use of social support principles. Theories of behavior change such as the Social Cognitive Theory and Self-Efficacy Theory were used often to underpin these systems. However, significant trends in the use of persuasive system features on par with behavior change theories could not be established from the reviewed studies. This points to the fact that there is still no theoretical consensus on how best to design interventions to promote behavior change in patients with CHD.

**Conclusions:**

Our results highlight key software features for designing BCSSs for the prevention and management of CHD. We encourage designers of behavior change interventions to evaluate the techniques that contributed to the success of the intervention. Future research should focus on evaluating the effectiveness of the interventions, persuasive design principles, and behavior change theories using research methodologies such as meta-analysis.

## Introduction

Coronary heart disease (CHD), also referred to as coronary artery disease (CAD), is the third leading cause of death worldwide and is associated with 17.8 million deaths annually [1]. Despite its significant association with a high mortality rate, it is preventable. With risk factors such as a sedentary lifestyle, physical inactivity, smoking, poor diet, hypertension, and obesity, both pharmacological and nonpharmacological interventions have been proposed to mitigate this menace [2]. Existing evidence suggests that preventing CHD requires lifestyle and health behavior changes [3]. Digital interventions, particularly behavior change support systems (BCSSs), have the potential to reduce risky health behaviors, improve the well-being of the user, and promote healthy lifestyles in patients with CHD. These are information systems that are designed to form, alter, or reinforce the attitudes, behaviors, or compliance of their users voluntarily [4]. A key element in behavior and attitude change is persuasion; the intention to change the behavior of an individual via persuasion may lead to a positive behavioral outcome [5]. Over the past decade, BCSSs and persuasive design have received elaborate attention with strategies that stem from behavior change theories. Although the development of these systems has increased at a startling rate to promote behavior change, the persuasion context (ie, the interdependencies between the user, technology, and the problem domain) and persuasive systems design principles are often ignored [6].

The existing literature reviews have predominantly focused on determining the effectiveness of different kinds of health BCSSs in changing lifestyle behavior, controlling modifiable risk factors, and improving CHD patient outcomes using mobile technologies [7,8], web-based technologies [9], and telerehabilitation [10]. However, evidence on how these systems were developed (ie, the design principles) to achieve the reported behavior change outcomes is not clear [11,12]. Moreover, these studies have focused on specific technologies; hence, the evidence cannot be generalized for all CHD BCSSs. Designing for behavior change involves identifying behavioral goals [5] and gaining an understanding of the behavior change context including the behavior change strategies, system features, and theoretical foundations [13,14] that underpin it.

This scoping review seeks to address this gap by providing an overview of persuasive context and behavior change strategies that support the management of CHD. Identifying these features will provide designers and researchers with an understanding of the persuasion context and persuasive features in systems that seek to promote behavior change in patients with CHD. More specifically, this review seeks to answer the broad review question, What persuasive systems design trends are evident in the management of CHD? To answer this question, this review aims to identify existing health BCSSs, report the characteristics of these systems, and describe the persuasion context and persuasive system design principles of the identified BCSSs for CHD using the Persuasive Systems Design (PSD) model proposed by Oinas-Kukkonen and Harjumaa [13].

The PSD model is the most-used framework for designing and evaluating persuasive systems [15]. Built on theories from psychology, information systems, and other disciplines, the PSD model guides the analysis of the persuasion context, including recognizing the intent of the persuasion, understanding the persuasion event, and defining the strategies in use [13]. Recognizing the persuasion intent involves understanding the roles of the persuader, the persuadee, the change type (ie, compliance, behavior, and attitude change), and the outcome (ie, forming, altering, or reinforcing compliance, behavior, and attitude change). Understanding the persuasion event entails features and characteristics arising from the problem domain (ie, use context), the user (ie, user context), and technology (ie, technology context). Persuasive systems have information content (ie, message) and software features. Defining the strategy involves crafting the content of the message to be delivered, deciding how to present arguments, the route to deliver the message, and the persuasiveness of the message. The model also provides a structure for designing and evaluating persuasive systems based on 7 key postulates and 4 system design principles, including primary task support (supports the user to carry out the actions that will lead to the desired behavior), dialogue support (facilitates the interaction between the user and the technology), credibility support (enhances the perception of trust and reliability of the system), and social support (aids behavior change by leveraging social influence). Table 1 describes specific variables of the PSD model used in evaluating and analyzing the persuasion context and system design features of the reviewed studies.

**Table 1:** Description of Persuasive Systems Design (PSD) variables used in the analysis, based on Oinas-Kukkonen and Harjumaa [13].

Factor					Description
**Analyzing the persuasion context**					
	**The intent**				
		Intended outcome/change	The intended outcomes of interest in this review will be classified as clinical outcomes, behavioral outcomes, psychological outcomes, and improved quality of life.		
		Designer/persuader bias	This refers to the unintentional influence that designers, developers, or creators have on the features, content, or functionalities of an intervention due to their viewpoints, experiences, or preferences.		
	**The** **event**				
		Use context	The general application domain is health—specifically, CHD^a^ as a preventable health condition.		
		User context	The population of interest will be patients living with CHD.		
		Technology context	This will include any technological platforms including wearable devices, mobile apps, and web apps.		
	**The strategy**				
		Message	This will describe the content delivered to inform or educate the user to change their behavior.		
		Route	This will describe how information and content are delivered to users via the direct route, which uses the user’s cognition, or the indirect route using societal cues.		
**Persuasive principles**					
	Primary task support			System features that support CHD to perform their primary task by reducing the cognitive load associated with the activity	
	Dialogue support			System features that provide computer-human dialogue as a means of reinforcing and motivating patients with CHD to perform the primary task	
	Credibility support			System features that make the patients with CHD believe that the intervention is reliable and credible	
	Social support			System features that motivate patients with CHD using social influence	

^a^CHD: coronary heart disease.

Drawing upon the PSD model, this research aims to provide an overview of BCSSs for managing CHD, report the characteristics of these systems, and describe the persuasion context and persuasive design principles. The rest of the paper is organized as follows: The Methods section describe how this research was conducted. The Results section reveals the findings of the research. This is followed by the Discussion section, which includes the implications of the findings and the conclusion.

## Methods

### Identification of Studies

This scoping review was conducted by following PRISMA-ScR (Preferred Reporting Items for Systematic Reviews and Meta-Analyses Extension for Scoping Reviews) [16]. We conducted a scoping literature search in the Scopus, Web of Science, and MEDLINE electronic databases from 2010 to 2022 using the Population, Intervention, Comparison, and Outcome (PICO) approach. The search domains included patients with CHD aged 18 years and older (population), randomized controlled trial (RCT) and BCSSs for CHD (intervention), and behavior change (outcome). The following search string related to the PICO approach was used (“coronary artery disease” OR “coronary heart disease” OR “ischemic heart disease”) AND (“mobile” OR “smart phone” OR “smartphone” OR “web” OR “internet”) AND (“prevention” OR “intervention”). The literature search was limited to studies published from 2010 because the PSD framework was proposed in 2009. Thus, including studies from 2010 would reveal evidence-based research trends. The first and second authors (EEYFA and AE, respectively) carried out the literature search and study selection independently. Divergent opinions on study inclusion were resolved through consensus among the 3 authors.

### Data Inclusion and Exclusion Criteria

The titles and abstracts were screened for keywords by the first and second authors. These authors downloaded the full text and examined if they were suitable using the following criteria. First, the study had to be an RCT on CHD, published in a peer-reviewed academic journal or conference, and written in the English language. Additionally, the study intervention had to be technology-mediated (ie, used mobile, web, or internet-based applications). Finally, the study intervention had to have the aim of promoting behavioral change, such as physical activity, diet, or smoking cessation to manage or prevent CHD.

### Data Extraction

Using the inclusion and exclusion criteria previously outlined, 30 papers were selected and reviewed. These articles were reviewed using the PSD framework. Data extraction and coding were conducted by the first and second authors independently. The 2 authors read the articles, identified the textual descriptions applicable to the design features, and coded them in a Microsoft Excel (Microsoft Corp) spreadsheet. The third author (HO-K) verified and validated the extracted data. Disagreements were resolved by revisiting the specific papers and reviewing them together until a consensus was reached.

The characteristics of the persuasion context and persuasive design features were extracted. The data extracted from each article were as follows: (1) name of the intervention; (2) objective of the study, which reveals the intention of the intervention; (3) primary and secondary outcome(s) (ie, intended outcome); (4) features/characteristics of the problem domain (ie, use context); (5) description of the study participants (ie, user context); (6) description of the technology-dependent characteristics of the intervention (ie, technology context); (7) description of the target behavior, and (8) persuasive software features of the intervention.

## Results

### Selection of Studies

**Figure 1:**
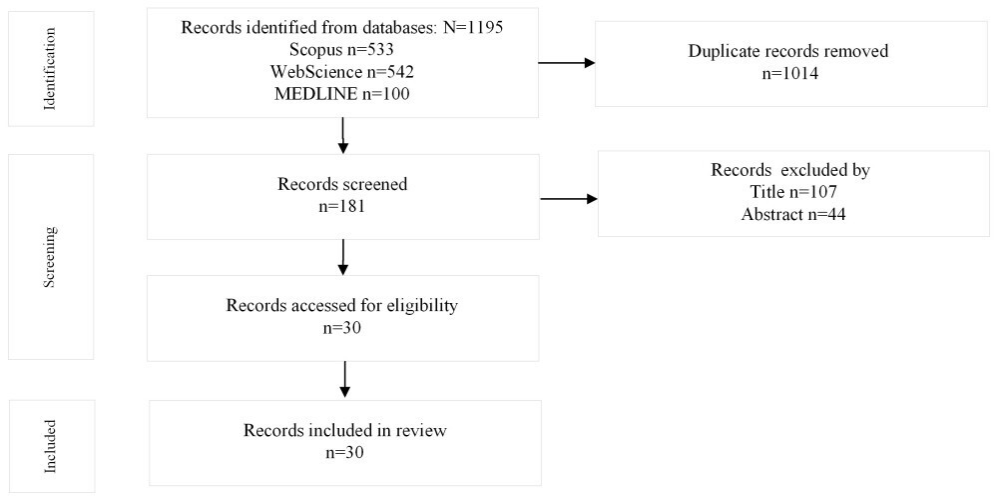
The PRISMA-ScR (Preferred Reporting Items for Systematic Reviews and Meta-Analyses extension for Scoping Reviews) flow diagram of the study selection process.

The initial search produced 1195 articles that were distributed among the aforementioned databases. Duplicates were removed, leaving 181 papers. The articles were excluded by title if they did not contain the keywords “CHD” and “randomized controlled trial, ” while articles were excluded by abstract if the technological context was not mentioned and if the intervention was not targeted at either behavior change or clinical outcome. Consequently, 151 articles were excluded by title and abstract. Finally, 30 RCT studies were analyzed in this scoping review. Figure 1 highlights the selection process using the PRISMA-ScR flow diagram.

### Characteristics of the Included Studies

The 30 included RCTs were published in 16 different peer-reviewed journals, namely *Journal of Medical Internet Research* (n=5, 17%), *JMIR MHealth and Uhealth* (n=4, 13%), *European Journal of Preventive Cardiology* (n=3, 13%), *Journal of Cardiopulmonary Rehabilitation and Prevention* (n=3, 10%), *Hearts* (n=2, 7%), *BMJ Open* (n=2, 7%), *Circulation* (n=2, 7%), and the rest in *JAMA*, *Lancet Digital Health*, *Plos ONE*, *Patient Education and Counseling*, *Journal of Cardiovascular Translational Research*, *Pharmacy Education*, *JAMA Cardiology*, *Coronary Artery Disease*, and *Health and Quality of Life Outcomes*.

The study duration for all included RCTs ranged between 4 weeks to 52 weeks, and the sample size for study participants ranged between 84 and 879. Additionally, 70% (n=21) of the studies focused on patients with CHD, 10% (n=3) on patients with acute coronary syndrome, 7% (n=2) on patients with CAD, and the remaining 13% (n=4) on patients with CHD and diabetes, CHD and depression, ischemic heart disease, and clinical manifestation of atherosclerosis in the coronary, cerebral, or peripheral arteries.

### Analyzing the Persuasion Context

#### Overview

As highlighted in Table 1, analyzing the persuasion context involves recognizing the intent of the persuasion, understanding the persuasion event, and defining the strategies in use.

#### The Intent

All studies stated the objective of their study, which reveals the intention of the persuader as well as the intention behind the intervention. The intention behind the interventions was to use information and communication technology to support patients with CHD to improve their health and lifestyle. The persuaders were researchers (ie, people who participated in the design), and in some cases, clinicians (when the study was intended to be used in a clinical setting). The persuadees were study participants who received the intervention in the RCT. In the design of the interventions, decisions regarding features and content were mostly influenced by the viewpoints, experiences, and assumptions of the design team. Only 17% (5/30) of studies involved users directly. These users were primarily involved in creating messages delivered through the intervention [17-21]. One (3%) study obtained feedback from users via a pilot test to improve the intervention [22]. Two (7%) studies disclosed the composition of their design team, yet users were not involved in the process [23,24]. Finally, 73% (n=22) of studies did not provide any information on the users/prospective users’ involvement in the design of the intervention. Given the potential effect of designer bias on the usability and effectiveness of an intervention, it is important to acknowledge this and put in measures to mitigate its effects.

**Figure 2:**
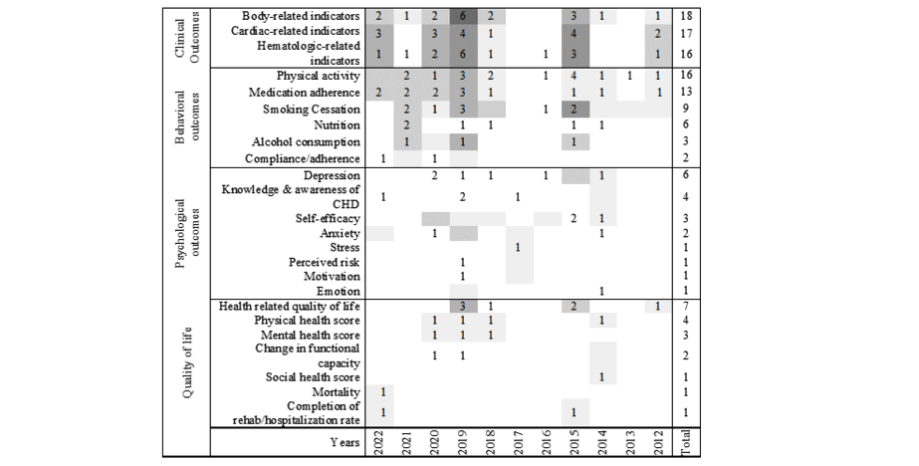
Distribution of intended outcomes. CHD: coronary heart disease.

We identified and classified the intended outcomes of the 30 included RCTs into 4 categories: clinical outcomes, behavioral outcomes, psychological outcomes, and improved quality of life. The distribution of identified intended outcomes in each publication year of the included studies is presented in Figure 2. Multimedia Appendix 1 contains more detailed information [17-46].

#### The Event

Analyzing the event provided insights into the problem domain (use context), user characteristics (user context), and technology (technology context). Although the main user group was people who had been diagnosed with CHD, some of them had multiple health conditions, such as diabetes and depression, or needed to change at least one behavior (eg, smoking, physical inactivity, and medication adherence) to prevent further health complications. The varying characteristics of the use context presented unique scenarios and opportunities for health behavior change (Multimedia Appendix 2 [17-46]). Users were mainly older than 18 years. A total of 20 (67%) studies [17-22,24,25,28,30,31,33,35-37,39,40,43,46] required the users to be able to read and understand the text, while 2 studies (7%) [18,29] required users to be computer literate.

The RCTs were conducted on various continents, including Oceania, Asia, Europe, and North America. In Oceania, studies were conducted in Australia [17,20,30,43,45,46] and New Zealand [26,33-35,38]. In Asia, there were 6 (20%) studies conducted in China [19,21,27,28,32,36,42], 3% (n=1) from Singapore [23], 3% (n=1) from the Republic of Korea [25], 3% (n=1) from Indonesia [39], and 3% (n=1) from Pakistan [31]. In Europe, 7% (n=2) of studies were conducted in the United Kingdom [18,40], 3% (n=1) in Italy [44], 3% (n=1) in the Netherlands [22], 3% (n=1) in Belgium [29], and 3% (n=1) across multiple European countries [41]. Two (7%) studies were conducted in North America, namely, the United States [37] and Canada [24].

Furthermore, it was observed that the nationality and cultural factors of users influenced the design of the intervention. For example, in Dorje et al [27], the popular Chinese social media app WeChat, together with Chinese avatars, was used to educate patients. Moreover, different types of technologies were chosen for the design and development for various reasons. The selection of the type of technology was based on some characteristics of the problem and user domain. For example, an SMS or other type of text message was used for delivering the intervention in one study because of its ease of use and cost-effectiveness [20]. In China, WeChat, which has an instant messaging component, was used in 3 (10%) studies [27,36,42].

#### The Strategy

Exactly 25 (83%) studies stated that they used content in their intervention. The content presented to the users in the 25 studies was mainly to educate users and support the behavior change process. This implies that the user’s cognition was required to process the information presented to them. Additionally, the RCTs conducted in Oceania and North America presented content to their users via the interventions, while 2 (7%) RCTs in both Asia and Europe did not present any content. Multimedia Appendices 3 and 4 [17-46] contain additional details.

Within the included RCTs, half (n=15, 50%) incorporated behavior change theories in their intervention designs. The most used behavior change theory was Social Cognitive Theory (n=10, 33%) [17,19-21,24,28,31,35,38,45]. This was followed by Self-Efficacy Theory, which was used in 17% (n=5) of studies [26,33,34,37,38]. Two (7%) studies [17,20], used the Behavior Skill Model, Theory of Reasoned Action, Theory of Planned Behavior, and Control Theory. The Health Belief Model was used in 2 studies [23,31] and the Commonsense Model was used in 2 studies [26,35]. Meanwhile, the Health Action Process Approach theory [28] and Cognition and Behavior theory [42] were each used once. It was observed that the number of theories used across these studies varied from 1 to 5. Interestingly, none of the RCTs conducted in Europe used behavior change theories in their interventions (Multimedia Appendix 4).

### Analyzing the Persuasive Features

#### Overview

System features that were used in the CHD interventions were identified and coded based on the PSD model software feature categories. As shown in Table 1, they comprised primary task support, dialogue support, credibility support, and social support. Multimedia Appendix 4 highlights the distribution of identified persuasive system features. Apart from Putra et al [39], all the analyzed interventions used a minimum of 1 persuasive feature and a maximum of 6 persuasive features. Features of the primary task support principle were the most used, while that of the social support principle was the least used in the interventions for patients with CHD (Multimedia Appendix 5 [17-46]).

#### Primary Task Support

Primary task support features assist the user in carrying out the primary tasks that lead to behavior change; they include personalization, self-monitoring, reduction, rehearsals, tunneling, tailoring, and simulation [13]. Personalization (n=23, 77%) and self-monitoring (n=18, 60%) were found to be the most widely represented primary task support features in the articles reviewed. Tailoring was identified in 6 (20%) studies. The reduction feature was identified in 1 (3%) study to grade tasks for users [18]. Features such as rehearsal, tunneling, and simulation were not identified. Personalization and semipersonalization were implemented in different forms, such as (1) selecting and providing educational content on CHD based on user characteristics, such as age and gender [40], and baseline characteristics [17,20,43], such as smoking status and diet; (2) using the preferred name of users [19,21,26,35,37] (semipersonalization); (3) using their smoking status and diet pattern [25] (semipersonalization); (4) sending messages at the preferred time of the user [26,35]; (5) individualized exercise programs [27,29,33,38,42]; (6) individualized feedback [28,45]; (7) customizable sounds for reminders [46]; (8) a personalized website based on risk factors [22]; and (9) personalized medication SMS text messages [31].

Self-monitoring was implemented by setting and tracking goals [18,24,40], monitoring physical activities [26,27,29,30,34,44], glucose monitoring [19], heart rate monitoring [27,42], monitoring of cardiovascular risk factors [45], blood pressure monitoring [21,32], step counting with a pedometer [26], and medication adherence using electronic pill bottles [37]. Different messages were tailored for different user groups. For example, different messages were delivered to smokers and nonsmokers [17,20,35,43], dietary messages for vegetarians and nonvegetarians [25] and tailored programs for the working population [23].

#### Dialogue Support

This support category comprises 7 system features, namely reminders, praise, rewards, liking, similarity, suggestions, and social role. Dialogue support incorporates forms of social or interpersonal interactions into feedback to encourage the user to respond to requests made by the intervention that may lead to behavior change [47]. Reminders, praise, rewards, suggestions, and social role were identified in the studies.

Reminders were the most used software feature in this category (n=13, 43%). Some examples of how reminders were implemented include reminders about behavior change to decrease CHD risk [17,20,43], exercise reminders [23,38], reminders to check blood pressure [27], push notifications [30], medication reminders [31,32,36,37,46], and checkup reminders [26]. This was followed by praise (n=12, 40%). The implementation of praise included individualized feedback to manage outcomes [27,28], motivational messages [29,33,41], personalized feedback on progress [33,40,42,45], and performance [18,26,34].

In addition to these, suggestions (n=3, 10%), rewards (n=10, 3%), and social roles (n=10, 3%) were used, albeit rarely. Suggestions were implemented by providing tips on overcoming hindrances [40], dietary recommendations [46], and information on various physical activities [33]. The social role feature was implemented via a virtual cardiologist coach who offered advice to users [27]. Reward in the form of social rewards was identified in the intervention by Devi et al [18]. Though the liking and similarity feature may have been present in the interventions, we could not evaluate it due to its subjective nature.

#### System Credibility Support

Design principles in this category support the credibility of the system as a function of persuasion. Its features include expertise, surface credibility, authority, third-party endorsement, real-world feel, trustworthiness, and verifiability. Here, the visual elements of the user interface, as well as the believability of the content of messages or information delivered via the intervention, are crucial. Expertise was evident in most of the interventions (n=10, 33%), as the content was created by experts. Multidisciplinary teams consisting of researchers, physicians, clinicians, therapists, and practitioners (ie, software developers) were involved in creating content for the interventions [17,19-21,23-25,31,36,43]. This was followed by authority (n=9, 30%) and verification (n=50, 17%). Authority was identified in several interventions [17,19-21,25,30,33,36,43]. This was in the form of citing health quotes from recognized authorities such as the National Heart Foundation of Australia and the American College for Sports Medicine. Verifiability was identified in 17% (n=5) of the interventions [22,23,25,27,33]. In these interventions, links were provided for fact-checking purposes. A total of 5 (17%) interventions were observed to implement the “real-world feel” feature such that participants could contact researchers or clinicians for various purposes [22-24,31,42].

#### Social Support

Social learning was the only social support feature identified in the analyzed interventions. The intervention enabled users to watch others go through a similar behavior change process [38]. Other features, such as social comparison, cooperation, social facilitation, normative influence, competition, and recognition, were not identified in the research articles. The minimal use of social support features may be associated with the tendency of these features to trigger high intensities of negative sentiment and emotional backfire [48,49].

## Discussion

### Principal Findings and Implications

CHD is a severe health problem, with an increasing prevalence worldwide. Adopting a healthy lifestyle and being aware of CHD risk factors are essential for its prevention and management. This scoping review identified existing health BCSSs from RCTs, reported their characteristics, and analyzed the persuasion context and persuasive design principles of the systems identified for CHD self-management using the PSD model. We found that trends in the use of persuasive system features on par with behavior change theories were identified for only 50% (n=15) of the RCTs; this points to the fact that there is still no consensus on the need to use theories and the best approaches to design interventions to promote behavior change in patients with CHD.

The intention behind the BCSSs was to support patients with CHD to improve their health and lifestyle. The interventions analyzed sought to improve clinical outcomes, behavioral outcomes, psychological outcomes, and improved quality of life outcomes associated with CHD for their users. The analysis of the events provided insights into the problem domain, user characteristics, and technology-related factors. Although the main user group was people who had been diagnosed with CHD, some of them had multiple health conditions such as diabetes and depression or needed to change at least one behavior (eg, smoking, physical inactivity, and medication adherence) to prevent further health complications. Users of the interventions were aged at least 18 years, cognitively sound, and computer literate.

Different types of information technology platforms were chosen for BCSS design and development for various reasons. For example, SMS and other types of text messages were used for delivering the intervention in 3% (n=1) of the studies because of their ease of use and cost-effectiveness. Moreover, WeChat, an instant messaging app, was adopted because it is a widely used social networking site in China.

Furthermore, the analysis of the strategy showed that the content delivered via the BCSSs was for educational purposes and hence required users to engage their cognitive resources. Another interesting finding was the lack of educational content in 13% (n=4) of the interventions. One would expect BCSSs to have educational content because such content provides a means to educate patients and reinforce behavior change [50].

Additionally, although the RCTs conducted in Europe used at least one persuasive feature in their interventions, none of the studies mentioned the behavior change theory that underpinned the BCSS. Proponents of the use of behavior change theories argue that theories explain the mechanisms through which behavior change occurs [51]. Often, developers prioritize the use of behavior change techniques at the expense of gaining an in-depth understanding of the theories that underlie them [14]. A potential problem that may arise with the use of BCSSs without a solid theoretical foundation is the creation of conflicting mechanisms that can affect the system’s long-term effectiveness. This also points to the fact that there is still no consensus on the relevance of theories and best approaches to design interventions to promote behavior change in patients with CHD.

Another issue worth mentioning is designer or persuader bias and how it manifests itself in designing persuasive and behavior change interventions. This bias may have important ethical implications, as persuasive design decisions should be neither deceptive, manipulative, nor coercive [52]—nor cause harm to the users. Although a lot of emphasis has been placed on user-centered design in recent times, there may be a tendency for designers and developers to make design decisions affected by cognitive biases (such as the inability to evaluate all solutions to determine optimal solutions due to resource limitations) and illogical decisions (eg, intuitive reasoning, which uses low cognitive resources) [53]. In the design of BCSSs, persuader bias can manifest itself in, for example, the kind of content presented to users and the implementation of features. Designers are tasked to make design decisions based on insights generated from the context of use, user characteristics, and affordances of technology [13]. These decisions can influence the acceptability and, subsequently, the effectiveness of the system because users are sensitive to design features (eg, tailoring) [54]. From the results of the analysis, it appears that the involvement of users (eg, to create messages) may serve as a strategy to mitigate designer bias in the development of health interventions. Further research is needed to confirm this claim.

The findings from this review suggest that BCSSs have the potential to support the self-management of CHD and promote healthy lifestyles. Studies argue that the effectiveness of these systems depends on their design and implementation [55,56]. The major challenge that emerges is identifying the components of the BCSS and pertinent PSD features that are responsible for driving behavioral change [56]. This will require a deep understanding of the intricate interplay between various elements within the BCSS, such as behavior change theories, educational content, and persuasive features. Additionally, knowing the individual contribution of persuasive components and synergistic effects in this regard is desired [56]. Further research shows that the patterns of use of the intervention influence its effectiveness in achieving the desired outcome [57].

Apart from Putra et al [39], between 1 and 6 persuasive features were used in the analyzed BCSSs. In the primary task support category, personalization and self-monitoring were found to be the most widely represented features. Within the dialogue support category, reminders and praise were the most-used features. Expertise and authority were the most-used features regarding credibility, while social learning was the only feature identified in the social support category. The frequent use of these features may imply their importance for BCSSs developed for managing or preventing CHD.

Additionally, we found that feedback is closely related to praise in the PSD model, yet distinct based on the textual descriptions. Users can receive feedback that is not necessarily of a positive tone (like praise). Feedback can be given on the user’s performance, which may be motivational, constructive, or both. Another interesting finding was the use of the term “semipersonalization,” a form of personalization. Semipersonalization describes a weak level of personalization. This brings to light the commentary by Oinas-Kukkonen [58] on a concept called “personalization myopia,” which seeks to clarify the misunderstanding surrounding the level and type of personalization offered in mobile and web apps. The level and type of personalization identified in the studies varied. In the studies analyzed, personalization of content was widely used. For instance, mobile text message–based interventions simply used the names of users to indicate personalization. With that being said, some of the studies did clarify the use of the level of personalization by using semipersonalization. A critical look at some studies revealed the use of user data to generate personalized content other than just the name. This is a step in the right direction toward true personalization. True personalization requires detailed information, such as the user’s preferences, to create individualized experiences [59] in the different stages of the user’s journey through the app. Moreover, the use of personalization accounts for differences in preferences between and within groups of participants [60].

It was reassuring that design principles that make the system credible were identified in 60% (n=18) of the BCSSs studied. System credibility support principles tend to increase user satisfaction and influence their intention to use an intervention [61]. Incorporating this principle shows the intention and commitment of the designers to deliver credible content and a trustworthy system.

### Limitations

We acknowledge some limitations in our work. First, we relied on textual descriptions provided in the original papers, which were subjectively interpreted by the reviewers, creating an avenue for subjective bias. Second, our study is based on English-language publications only, thus excluding publications in other languages that could provide rich content to this analysis. Third, this review did not analyze the effectiveness of the analyzed interventions as it fell beyond the scope of this research. Instead, the focus was on identifying trends in persuasive design characteristics used in BCSSs for CHD. This encompassed key persuasive elements like behavior change theories, techniques, educational content, and persuasive features in the analyzed studies. Also, recognizing prevailing trends can contribute to the refinement and development of theories and practices for BCSSs. This valuable information can also help developers, clinicians, and developers to make informed decisions. To deepen our understanding, future research should assess the relevance and impact of persuasive elements through meta-analysis. This approach may yield much-needed evidence and insights and contribute to developing effective interventions.

### Conclusion

This study sought to identify persuasive design characteristics in mobile, web, and other information system interventions for CHD. We analyzed 30 peer-reviewed RCT papers that implemented BCSSs for patients with CHD. This study highlights the key issues that should be considered when designing and developing BCSSs for CHD. From the analysis of RCTs, we found that trends in the use of persuasive system features on par with behavior change theories were identified for only 50% (n=15) of RCTs. This points to the fact that there is still no consensus on the need to use theories and the best approaches to design interventions to promote behavior change in patients with CHD. Although we were able to highlight the trends in the persuasive features, we were unable to determine if these features influenced the effectiveness of the analyzed BCSSs. Moreover, there is a need to evaluate the actual effect of the intervention on users. Thus, future research should address this using data analysis methodologies such as meta-analysis.

## Appendix

Multimedia Appendix 1 The analysis of intent describes the primary and secondary outcomes of the randomized controlled trials.

Multimedia Appendix 2 The analysis of the event describes the use, user, and technology context in the studied interventions.

Multimedia Appendix 3 The analysis of the strategy describes the message and route for presenting information in the studied intervention.

Multimedia Appendix 4 Distribution of identified persuasive systems features, contents, and theories as shown by the shaded areas. "Content" refers to the number of contents identified in each intervention. "Theories" refers to the number of theories identified in each intervention. AUT: authority; CRED: credibility support; DIAL: dialogue support; EXP: expertise; PER: personalization; PRA: praise; PRIM: primary task support; RED: reduction; REM: reminder; REW: rewards; RWF: real-world feel; SMO: self-monitoring; SOCI, SLE: social learning; SRO: social role; SUG: suggestion; TAI: tailoring; VER: verifiability.

Multimedia Appendix 5 The analysis of persuasive features used in the interventions examined.

Multimedia Appendix 6 PRISMA checklist.

## Abbreviations

BCSS: behavior change support system
CAD: coronary artery disease
CHD: coronary heart disease
PICO: Population, Intervention, Comparison, and Outcome
PRISMA-ScR: Preferred Reporting Items for Systematic Reviews and Meta-Analyses Extension for Scoping Reviews
PSD: Persuasive Systems Design
RCT: randomized controlled trial

## Data Availability

The data used in this research is publicly available in the Multimedia Appendices.
